# "Those who died are the ones that are cured". Walking the political tightrope of Nodding Syndrome in northern Uganda: Emerging challenges for research and policy

**DOI:** 10.1371/journal.pntd.0007344

**Published:** 2019-06-20

**Authors:** Julia Irani, Joseph Rujumba, Amos Deogratius Mwaka, Jesca Arach, Denis Lanyuru, Richard Idro, Rene Gerrets, Koen Peeters Grietens, Sarah O’Neill

**Affiliations:** 1 Department of Public Health, Institute of Tropical Medicine, Antwerp, Belgium; 2 Department of Pediatrics and Child Health, College of Health Sciences, Makerere University, Kampala, Uganda; 3 Department of Medicine, School of Medicine, College of Health Sciences, Makerere University, Kampala, Uganda; 4 The Ugandan Ministry of Health, Kampala, Uganda; 5 The Amsterdam Institute for Social Science Research, University of Amsterdam, Amsterdam, Netherlands; 6 CRISS, Ecole de Santé Publique and LAMC, Faculté de Philosophie et Sciences Sociales, Université Libre de Bruxelles, Brussels, Belgium; Ludwig-Maximilians-University, UNITED STATES

## Abstract

**Background:**

Nodding Syndrome was first reported from Tanzania in the 1960s but appeared as an epidemic in Northern Uganda in the 1990s during the LRA civil war. It is characterized by repetitive head nodding, often followed by other types of seizures, developmental retardation and growth faltering with onset occurring in children aged 5–15 years. More than 50 years after the first reports, the aetiology remains unknown and there is still no cure. The recent hypothesis that Nodding Syndrome is caused by onchocerciasis also increases the relevance of onchocerciasis control. Northern Uganda, with its unique socio-political history, adds challenges to the prevention and treatment for Nodding Syndrome. This article aims to show how and why Nodding Syndrome has been politicised in Uganda; how this politicisation has affected health interventions including research and dissemination; and, the possible implications this can have for disease prevention and treatment.

**Methodology:**

Ethnographic research methods were used triangulating in-depth interviews, focus group discussions, informal conversations and participant observation, for an understanding of the various stakeholders’ perceptions of Nodding Syndrome and how these perceptions impact future interventions for prevention, treatment and disease control.

**Principal findings:**

Distrust towards the government was a sentiment that had developed in Northern Uganda over several decades of war and was particularly linked to the political control and ethnic divisions between the north and south. This coincided with the sudden appearance of Nodding Syndrome, an unknown epidemic disease of which the cause could not be clearly identified and optimal treatment had not clearly been established. Additionally, the dissemination of the inconclusive results of research conducted in the area lacked sufficient community involvement which further fueled this political distrust. Disease perceptions revolved around rumours that the entire Acholi ethnic group of the north would be annihilated, or that international researchers were making money by stealing study samples. This discouraged some community members from participating in research or from accepting the mass drug administration of ivermectin for prevention and treatment of onchocerciasis. Such rumour and distrust led to suspicions concerning the integrity of the disseminated results, which may negatively impact future disease management and control interventions.

**Conclusions and recommendations:**

Trust must be built up gradually through transparency and by de-politicising interventions. This can be done by engaging the community at regular intervals during research and data collection and the dissemination of results in addition to involvement during service delivery for prevention and treatment. Maintaining a regular feedback loop with the community will help control rumours, build trust, and improve the preparations for adequate dissemination.

## Author summary

Nodding Syndrome, a form of epilepsy that occurs in onchocerciasis-endemic areas has affected about 3000 children in Northern Uganda where the epidemic emerged at the time of the civil war. Although an association with onchocerciasis has been established, the disease etiology has not yet been identified. In conjunction with the area’s history of war, political conflict and ethnic divides, many affected people were suspicious of government health interventions and scientific studies. Furthermore, ambiguities around the disease etiology leaves room for several interpretations, which in addition to distrust leads to rumours, that may hinder future interventions. In view of improving adherence to health interventions, this article explains the socio-political nature of Nodding Syndrome in Northern Uganda. We show how and why this disease has been politicised in Uganda; how this politicisation has affected research and dissemination; and, the implications this has for disease control. The insights presented will be critical for reducing resistance to research, to treatment and to the dissemination of knowledge which is necessary for the prevention and control of Nodding Syndrome and onchocerciasis in Northern Uganda.

## Introduction

Nodding Syndrome (NS), a severely debilitating neurological syndrome mainly affecting children, is considered a “political” issue in Uganda, unlike other affected areas such as Tanzania [1]. This “politicization” of NS is the result of a long history of ethnic divisions [2], which are tied up with political power struggles and the civil war that was ongoing at the start of the epidemic [1, 3]. Around 1998, when the war between the Lord’s Resistance Army (LRA) and the current government was at its peak, more than half of the population in northern Uganda was living in internally displaced persons [4] camps [3, 5]. This was when the NS epidemic erupted in Kitgum and neighbouring districts of Northern Uganda. The peak of the epidemic occurred in 2008 [5], two years after the Lord’s Resistance Army signed a truce with the Sudanese and Ugandan governments. People had just returned to their villages from IDP camps [5, 6] and were in the process of rebuilding their livelihoods after 10 years.

NS is characterized by repetitive head nodding, often followed by other types of seizures, developmental retardation and growth faltering with the onset occurring in children aged 5–15 years [7, 8]. The case definition of NS categorized as suspected, probable and confirmed cases, was agreed on at an international scientific meeting organized in Kampala by WHO in 2012. It is based on major criteria such as repetitive involuntary dropping of the head in a previously normal person and onset between 3 to 18 years in combination with one minor criteria such as other neurological abnormalities, temporal and spatial clustering, seizure being triggered by food or cold weather, stunting, delayed physical development and psychiatric symptoms (WHO 2012). As studies are producing more knowledge the case definition continues to be debated and modified. Symptoms of head nodding were first reported over 50 years ago in Southern Tanzania [9], and later in Liberia [10], South Sudan [11, 12] and Uganda [7]. There is still however, no clear biomarker for NS and the cause remains unknown despite a consistent epidemiological association with onchocerciasis, a parasitic infection transmitted by the blackfly [7, 13–15]. On-going research is exploring whether NS could be an auto-immune reaction to *Onchocerca volvulus (OV)[16, 17]*, the parasite causing river blindness [18] or an *OV-*induced neuro-inflammatory disorder [19] or a post-measles brain disorder triggered by malnutrition [20] or an autism spectrum disorder [21]. Another recent study suggests that NS in Uganda is a neurodegenerative disease [22]. Based on the spatial and temporal clustering of NS along with other epilepsies, some researchers are also speculating whether NS is just one type of epilepsy, occurring within a spectrum of epilepsies, observed exclusively in onchocerciasis-endemic areas to the extent that it has been proposed to move the focus from NS to ‘onchocerciasis-associated epilepsy’ [23]. This putative link increases the relevance of onchocerciasis control [24] for prevention and control of NS. While NS appears to be a form of epilepsy for which the cause remains unclear and the classification using the current case definition has proven to be problematic in the field-context and without highly sophisticated equipment, for this manuscript we refer to NS as a specific form of epilepsy that has an epidemiological association to onchocerciasis. The NS cases presented in the results are simply self-reported cases. The cases were not examined by a medical doctor for study and have not been cross-checked with patient data.

Onchocerciasis control programs in Northern Uganda began in 1994 but several years of civil conflict in the north have caused disruptions to these initiatives in the area [25]. However, since 2012, ivermectin (IVM) has been distributed bi-annually and rivers are treated with larvicides, to prevent onchocerciasis and other parasitic diseases in Northern Uganda [25]. Uganda has successfully eliminated onchocerciasis in all but two regions, one of which is the Madi-Mid North focus in Northern Uganda. The Mid-North focus includes Amuru, Gulu, Kitgum, Lamwo, Nwoya, Oyam, and Pader districts [25], where we also find NS. Since 2012, anti-epileptic medication is offered to NS patients free of charge among NS patients along with psychosocial care and nutritional supplements in government health centres and hospitals and by some non-governmental organisations (NGOs) [26]. While such symptomatic treatment of NS is not curative, it has been shown to help suppress seizures [27].

NS also negatively affects families socially and economically. Affected children need close monitoring in order to prevent them from running away or having serious accidents like falling into fire or drowning. Due to this, caretakers have to stay at home and are either unable to cultivate as much or are forced to tie children to trees in order to keep them safe [28]. Social isolation of the affected children is also common because community members believe NS is communicable [28].

This article presents the socio-political context of NS health interventions; in particular, how and why NS has been “politicised” in Uganda; how this “politicization” has affected research, and its dissemination and the implications this has for disease control. The insights presented will be critical for reducing resistance to research, interventions, treatment uptake and dissemination, which is necessary for the prevention and control of NS and onchocerciasis in Northern Uganda.

## Methods

### Study site and population

Ethnographic research was carried out in Northern Uganda between 2015 and 2017, primarily in Kitgum district and partly in Gulu district. These districts were selected purposively on the basis of their having NS affected and non-affected villages. In addition, the research team was introduced to these districts during the second NS conference held in Gulu and thus leveraged on linkages with district officials and Hope4Humans to focus the study in the two districts. Extending the study to other districts like Pader and Lamwo with similar ethnic composition as the study areas was not feasible. However, focusing on the two districts enabled us to visit the research subjects repeatedly over the three-year period (2015–2017) which facilitated gaining community trust and an in-depth understanding of the context and beliefs around NS.

The recent history of Northern Uganda, and our field sites, was dominated by the war between the Lord’s Resistance Army (LRA) and the government of Uganda, which lasted from 1987 to 2006. The forced displacement of much of the population into IDP camps took place from 1996–2007 where people were subjected to violence and governmental surveillance [29, 30]. Prior to the LRA war, Northern Uganda had endured over three decades of civil conflict, which enhanced the already existing North-South divide between the Acholi and Langi ethnic groups of the North and the Bantu in the South [31].

Political distrust and suspicion about perceived neglect by the current government towards the Acholi people is a recurrent sentiment that has prevailed through all three epidemics–Ebola, HIV [32] and now NS. President Museveni came to power by overthrowing an Acholi leader, General Tito Okello Lutwa in 1986. As a counterinsurgency, the LRA war against president Museveni and his National Resistance Army [33] began a year later, mainly in response to alleged atrocities by the NRA against the Acholi who had feared retribution by the new government [32]. These feelings have been reinforced by the level of economic investment, governmental and non-governmental development initiatives, social services and business-opportunities available in the south of the country as compared to the substantially less developed northern region [32]. When responding to disease outbreaks in Northern Uganda it is crucial to keep this historical political context in mind.

In 2015, a study retrospectively reported that the first case of NS was seen in Western Uganda in 1994 [34], however, it was only after media uproar in 2012 that NS attracted national attention through a campaign of the opposition party [3] highlighting the perceived neglect of healthcare in the North [3, 35]. Consequent parliamentary debates about the outbreak of a previously unknown ‘disease’ named “Nodding Syndrome” drove governmental and non-governmental organisations (NGOs) to provide services and resources for NS control [3]. These consisted of free of charge provision of anti-epileptic drugs (AED) for the management of seizures, along with nutritional supplements as well as behavioural and physical therapy at 17 government health facilities in the affected region [19]. However, the resources provision for the NS response have been inadequate for different reasons.

Between 2012 and 2017, NS outreach services and comprehensive in-patient care for severely affected children were also provided by a US funded NGO, Hope for Humans in Gulu district. In late 2017 the NGO had to close down after several failed efforts of finding a sustainable stream of funding or governmental support. This closure led to renewed accusations and media reports of the government’s and ministry of health’s failure to prioritize and protect Acholi children. After much political debate the new government committed to improved NS service provision in Northern Uganda. There has been a cyclical trend of political propaganda, where the Acholi in the north, both politicians and civilians, have criticized the ruling government of inadequate support, which has driven governmental interventions for NS in the region.

The NS epidemic affected an already impoverished region of the country, exacerbating the economic burden on caretakers and the community [28, 36]. The region is still one of the most impoverished regions of the country. While 85% of Uganda’s poor were reported to live in the Northern and Eastern regions of the country in 2013, the Northern region accounted for more than half [37]. The villages we visited in Gulu and Kitgum districts, survived mainly on subsistence farming. Droughts and el niño rains periodically affected the food production and inhabitants faced severe food shortages.

Village inhabitants were mostly of the Acholi ethnic group, who had lived in the area for several generations but were displaced from their homes during the LRA war, when they lived in IDP camps [29].

### Sampling

Contrasting villages with high prevalence (more than 6%) and low prevalence (about 1%) of epilepsy/Nodding syndrome were purposefully selected. Prevalence was calculated based on the 2012 data provided by the ministry of health for all villages in the Kitgum district, and from the Hope4Humans estimates and the local health centre for Gulu district. The study began with an exploratory phase, where data was collected from two high prevalence (Akoyo and Ajan) and one low prevalence (Dino) village in Gulu district, and from three villages in Kitgum district. Health officials at the district hospital which had a ward that handled epilepsy and nodding syndrome cases were interviewed. The main phase of data collection focused on Kitgum district as we were able to find 10 contrasting villages within two sub-counties, Akwang and Amida, which made it relevant for our study. Villages selected in Kitgum district are listed in Table 1. All villages were classified as hyper-endemic for onchocerciasis.

**Table 1:** Prevalence of Nodding Syndrome & epilepsy in selected villages in Kitgum district.

District	Sub-county	Parish	Village	Prevalence classification as per 2012 data
Kitgum	Akwang	Lamit	Tumangu	high
Kitgum	Akwang	Pajimo	Bola	high
Kitgum	Akwang	Lamit	Adyee	high
Kitgum	Amida	Okidi	Awere	high
Kitgum	Akwang	Lamit	Labworomo	high
Kitgum	Akwang	Lugwar	Gogo	low
Kitgum	Akwang	Lamit	Liba	low
Kitgum	Amida	Koch	Tai Ocot	low
Kitgum	Akwang	Pajimo	Abudere	low
Kitgum	Amida	Lukwor	Opette	low

Participant recruitment was carried out through a mixed-sampling approach, including both theoretical (based on emergent findings) and snowball sampling techniques (relying on one sampled person to contact the next person in the sample) in order to have maximum variation and representativeness in the sample. We purposefully selected varying social groups based on age; gender; persons directly affected/unaffected by NS/Epilepsy; occupation and livelihood regardless of local hierarchy or locally perceived expertise as shown in Table 2.

**Table 2:** Respondents.

	TOTAL	Prev 6% or more	Prev 1% or less	Mixed
**Total FGD**	**13**	**8**	**4**	**1**
FGD Mixed (male/female, affected/unaffected)	6	4	2	0
FGD Women only	4	2	2	0
FGD Health staff—village health workers	1	0	0	1
FGD Caretakers only	1	1	0	0
FGD Teachers	1	1	0	0
**Total IDI**	**94**	**67**	**27**	**-**
IDI—Caretaker	30	24	6	
IDI—Community member (unaffected)	22	17	5	
IDI—Health staff	23	14	9	
IDI—Politician	4	2	2	
IDI—Welfare/social worker	2	2	0	
IDI—Traditional healer	3	2	1	
IDI–Individuals with NS/Epilepsy	8	6	2	
IDI—Teacher	2		2	

### Data collection

An ethnographic approach, triangulating participant observations, in-depth interviews, focus group discussions, was chosen because it allows in-depth descriptions of disease perceptions and the detection of unforeseen and unknown variables that are hard to measure quantitatively [38]. Additionally, text analysis of media reports was used to deepen the contextualization of the political environment and cross-check emerging themes. Data collection centred around emergent themes such as perceptions of epilepsy and the experiences of those affected, access to and perceptions of public, private and traditional health facilities in general and specifically for NS/epilepsy, health seeking behaviour, religious practices, daily activities and livelihoods.

Participant observation was used to acquire an understanding of the local context. The researchers participated in daily activities in the community and the home setting, observed and participated in events in their usual context, while having informal conversations with a variety of community members. This method was used to cross-check the validity of information obtained in semi-structured interviews by establishing rapport and building trust with community members to reduce response bias. Repeated visits were made to the selected villages over the course of two years. Brief notes were taken during informal interviews if appropriate, otherwise the conversations were written down in detail afterwards.

94 in-depth interviews were conducted at participants’ homes in private or in places where the respondent felt at ease. Most in-depth interviews took an hour and were audio recorded with consent and conducted in the local language, Acholi. If the respondent did not wish to be audio recorded, detailed hand-written notes were taken. Later, all recordings were transcribed verbatim in Acholi and translated in English by trained field assistants.

13 formal focus group discussions were conducted (12 in Kitgum and 1 in Gulu) mostly at village centres, at market places or outside people’s homes when people were gathered together to socialise or for an event or they were organised by the village leaders in advance. Group discussions were used for different purposes: i.e., to get a quick picture of the village activities, livelihoods and facilities available like health centres, schools, traditional healers, water access points, and with village elders to get a better idea of how things might or might not have changed in contrast to the past, with the same gender to understand social norms, gender roles and disease perceptions. Some group discussions were audio recorded with consent while some were documented with hand written notes. All discussions were conducted in the local language, Acholi. Like IDIs, all recordings were transcribed verbatim in Acholi and translated to English by trained field assistants.

Articles in media were monitored from 2015–2017 by using ‘google alerts’ service, i.e. a notification tool offered by Google based on set keywords, for the keywords ‘nodding disease’ and ‘nodding syndrome’ respectively. Most articles came from online English newspapers of Uganda, like “The observer”, “The monitor”, “Daily monitor” and “Acholi times” and occasionally there were some from “Huffington post” and scientific journals. Earlier articles dating back to 2012 were searched for through a google search. All articles were used to assess what was being presented in the media and analysed comparatively with emerging themes from the field.

### Data analysis

Data analysis was retroductive (combining inductive analysis of field data and theory from existing anthropological literature)[39] and was carried out concurrently with data collection. Relevant categories for analysis were identified combining data from participant observation, interviews and group discussions. Through the analysis process, data were constantly subjected to theoretical perspectives so as to ensure theoretical triangulation and to embed the findings in existing literature. We used concepts of uncertainty and meaning making coupled with sense making of epilepsy as elaborated by Susan Whyte [40, 41]. We also looked at fear and distrust and its role in the creation of rumours [42], also in conjunction with other epidemics that occurred in the same area to conceptualise the unique socio-political context [32, 43, 44], annihilation anxieties of minority and marginalised populations [45]. Data were imported, managed and analysed using NVivo 12 Qualitative Data Analysis software (QSR International Pty Ltd. Cardigan UK).

### Ethical considerations

The study was reviewed and approved by the Institutional Review Board of the Institute of Tropical Medicine in Antwerp (IRB/AB/ac/036 Ref: 983/14); the Makerere University School of Medicine’s Research Ethics Committee (REC REF 2015–079) and the Uganda National Council for Science and Technology (REF: SS 3845). Oral consent was obtained from all participants and preferred, since the act of signing one’s name when providing information can be a reason for mistrust, particularly among illiterate populations, which can significantly reduce the quality of the data (Refer to justification of oral consent attached). Oral consent was documented by the interviewer on a separate paper form. Interviewers followed the European Guidelines for FP7 projects and the guidelines of the American Anthropological Association.

Children who are able to give assent to participate in research were asked to provide assent in addition to the consent of the legally authorized representative. Young people aged 16–18 with sufficient understanding were asked their full consent to participate in research independently of their parents and guardians. If a potential respondent was cognitively impaired, a close family member was asked for consent to ask questions pertinent to the potential respondent. Confidentiality was maintained by storing all data on a password protected computer and not transcribing names of respondents. Findings from the study will be disseminated in the form of scientific manuscripts which will be shared with the Makerere University School of Medicine’s Research Ethics Committee and the Uganda National Council for Science and Technology and the Ministry of Health.

## Results

### Making sense of a ‘new’ disease in a politically contested, war-torn area

Given the timing of the NS epidemic, community members linked the aetiology of NS to the LRA war in several ways. The most commonly perceived cause was poisoning either through the pollution of air and water with gunpowder during the war or by toxins in the food distributed at IDP camps. The second most commonly stated cause was that the spirits of the deceased during the war were angry for not getting a respectful burial and so were taking revenge on their children by inflicting this disease on them. Illness interpretations also included perceptions related to the epidemic occurrence of the disease outbreak. Crowding was seen to have enhanced the spread of the disease, ‘*like measles*, *through the air*’ due to the high concentration of people in the camps. The perceived epidemic nature of NS also led to some not believing that it was a form of epilepsy, an illness well known to them as illustrated in the following quote: “*You researchers say this is epilepsy*, *but we know epilepsy … we used to have it before but it was only one or two persons in the entire village who were affected… but this is different*. *This came during the war*, *and this affects entire villages*. *This is not epilepsy*, *this is because of the war*. *(…) Why is it that the villages where most atrocities took place*, *are also the villages that have the most people affected (by NS)*?*”* (*IDI*, *father of NS victim*).

Some associated NS with changes in political power, as illustrated at the funeral of an NS victim: “*During Obote’s first regime*, *there was a disease called ‘two nailon’ [gonorrhoea]*. *Later he was over thrown by Amin and this sickness disappeared … When Obote came back to power again*, *there came HIV/AIDS and this continued in Museveni’s regime*. *And in Museveni’s government there comes this sickness*, *this strange disease called nodding syndrome*.” (Sermon of a middle-aged Acholi Catholic priest)

Many respondents had also heard about the blackfly as a potential cause from researchers and health officials. However, few agreed with this possibility. Many were not convinced as they logically reasoned that blackflies had always been around while NS was something new that had started occurring during the war.

Some resented and distrusted the government and suspected they were the reason for this illness. This distrust was due to the perceived lack of response to the NS outbreak until 2012—almost 15 years after the first cases were noticed, and approximately six years after the war had ended. The government response was perceived as having been initiated after the political propaganda around the disease began circulating through the media attention.

Besides the growing sense of suspicion towards the government, Box 1 conveys the sentiment that governmental authorities did not want affected locals to say that NS existed and was increasing. This resentment explains the second uproar in the media in 2018, after the closing of the NGO, Hope for Humans. Hope for Humans was requesting governmental funds and encouraging the government to take over the responsibility. When this request was refused the NGO was unsustainable and closed down. It was evident that people felt that the government and the MoH were not acknowledging the severity of the problem. The media reported that there was an angry backlash from the northern political representatives when the MoH suggested that there were no more new cases of NS while their people strongly asserted that there were in fact new cases. While the MoH may not be wrong to say that there were no new cases of NS according to case definitions, community members still perceived all epilepsies that occurred during or after the war to be NS and thus thought that the MoH was covering up facts and dismissing the problems people in the north were facing.

**Box 1. *Delayed response and political propaganda driving initial response***

A middle-aged male village health worker, Orach, lamented about the first three cases seen in 1998. He said when they first noticed this strange disease, he took the children to the health centre in Kitgum which referred them to the hospital whose fees they could not afford, and so no action was taken. A few years later, his own son also got NS and he actively tried everything to get care. He said that it was only in 2012, after the Kitgum district’s parliamentarian from the opposition party voiced people’s suffering that they were taken to Kampala, the capital city, for treatment.

In an informal conversation, the parliamentarian explained that she highlighted the current leadership’s neglect of the children in the north and mobilised a bus full of 24 patients and parents to be taken to the main referral hospital in the capital city. She notified the president and the hospital staff that these children were on their way to be examined and that the children of the north should not be neglected. Orach was one of the parents on this bus with his son. Another parent, Atim, who was also on the bus explained that while on the way, the bus was intercepted by police and they were detained for two days without a reason. The police then escorted the parents and the 24 affected children to the hospital. At the hospital, the president met them and the media was there taking photographs of their children with the president. They were provided with meals and accommodation for the rest of their stay. This made parents suspicious once again and they wondered why the police were involved. “*We asked ourselves*, *‘what is the government trying to hide*?*’*”

… After treatment at the referral hospital, Orach said that the children showed improvement. However, once they returned to their home villages, they could not be fed in the same way and the children’s condition deteriorated. A year later, his son died reportedly due to uncontrolled, repetitive seizures. He said that researchers took a sample of his deceased son’s brain for testing but he had not heard back from them three years later (2015).

In 2017, when we met him again, he expressed distrust in the governmental system. He said, “*In the district here*, *(Kitgum) they don’t want us to say that such a disease exists or that it is increasing”*.

The political propaganda that drove the initial response to the outbreak influenced how NS became a tool for power both for politicians and for people in the community gaining resources through the epidemic. Box 2 shows that when these services were abruptly stopped, feelings of resentment and distrust towards the government were reinforced.

**Box 2. *Inconsistent interventions and suspicion***

A psychiatric nurse at the government hospital in Gulu, who was part of the initial interventions to address NS explained that after the political and media attention that NS received, outreach programs and clinics were established in 2012 by the government. There were severe food shortages at the time generally as people were slowly recovering from the war, and households with affected children were struggling even more. To cater for this, the government introduced a program according to which the households of the affected children were supplied with food along with the treatment for seizures free of charge. She said, “*NS is highly politicized so the community thinks that it is beneficial to have a child with NS*. *As people and the government are providing food*, *clothes*, *and money to these families*, *the Ministry of Health’s focus is on identifying Nodding properly because many people were claiming to have NS (when they did not have NS)*. *When this programme proved unsustainable*, *the government suddenly stopped providing food to NS families*. *There was no proper way of weaning them off*”. Consequently, the affected parents felt that “*the government does not care about us*”.

However, some still had a different view of the government. While most community members were disheartened by the prospects of NS and felt angered, frustrated and neglected by the government, some believed that researchers coming to the area were government agents who were not trying to find a remedy for NS.

### Consequences for health interventions

The perceived link between NS, the war, forced displacement to government-protected camps, in addition to the overwhelming burden of caretaking under extreme poverty, the social inequality and ethnic tensions all enhanced distrust of the government. These factors directly created barriers to NS service delivery in terms of limiting care available in the private sector, increased distrust and reluctance to accept treatment and prevention for *onchocerciasis*, *all relevant for managing NS*.

Some non-governmental health facilities, for example, the local Catholic missionary St. Joseph’s hospital in Kitgum, prefered not to treat NS patients due to the political nature of the disease. Reportedly, during the initial period when NS was getting political and media attention, the missionary hospital hosted a researcher who independently observed NS without informing them. After visiting one of the affected villages by himself, news reached the authorities and police tracked him down for questioning. The foreigner was reportedly arrested and deported for not following proper protocol and hospital employees were questioned. Whatever the veracity of the details of this and similar stories, it has led to sensitivity around accepting NS patients as illustrated by the following quote: “*We care for epileptic patients in this facility but for NS patients*, *we always refer them to Kitgum Government Hospital because NS has ‘political affiliations’*, *and we are faith-based so we don’t want to mix politics and religion*.” (*IDI*, *hospital staff*) What is interesting however, is how NS is distinguished from other epilepsies at hospitals, where NS and epilepsy were considered two separate medical conditions, whereas people in the community, perceived all epilepsies that occurred during or after the war as NS.

Additionally, news of incidents like this spread through the community quickly and like any story travelling through the grapevine, it lost or enhanced some details allowing differing interpretations to arise. Some became suspicious of secrecy they observed or by the hidden agendas of researchers, the government or both.

Despite the distrust of the government, most NS affected families still sought care in government facilities. While this behavior was seemingly counterintuitive, there were two main reasons for people to do so. Firstly, people were desperate for a cure as the burden of this disease was immense. Besides biomedical treatments, desperation had driven people to seek additional treatment options, including an array of traditional remedies through herbalists, spiritual healers and religious healers. This desperation was illustrated by a father who lost his son to NS. He expressed emphatically, “*Those who died are the ones that are cured*” implying that death among children with NS was often perceived as better than the continued suffering and disease-related hopelessness associated with NS within the context of poverty. Anti-epileptic drugs, when available, helped suppress symptoms for most patients and when the perceived benefit from them was greater than the cost, they continued to take the medication. A positive aspect of the biomedical health interventions in the area was that they were run and staffed by locals of the same ethnic group. So, while the community did not trust the government at large, they did trust the health professionals employed on a personal level. However, we also encountered a situation where one individual who had grown up in one of the villages there and came from a modest background, later was perceived as arrogant and driven by financial pursuits by community members after becoming successful. This individual was often used for sensitization by the MoH/government, which backfired as people did not trust in him despite their shared background and family history.

While exploring community perceptions towards MDA and the distribution of Ivermectin, a drug distributed to treat and prevent onchocerciasis, the general impression was that more people were taking IVM now as compared to when it was first introduced. In addition to continuous sensitization, the research showed that people got a sense that the medication helped relieve symptoms and there was a better understanding of the side effects and why they occurred. However, some were still refusing to take the medication. In addition to common fears of side-effects, one reason for refusing up-take was that they did not trust the purpose of the medication. Some households perceived it to be a government strategy to reduce the population in the region. The village health worker who distributes IVM said, “*People argue that yes*, *this government seems to be having a plan to finish off the people of northern Uganda*. *Why is it that they are bringing these drugs here and not to his (current leader*, *Museveni’s) village*?” (IDI, female, ivermectin distributor)

### Consequences for research activities

Due to the spatial and temporal clustering of NS, most studies have been conducted in a limited number of villages since the war ended. The village of Tumangu, for example, in Kitgum district is one of the most popular sites for research because it has had a high number of cases according to health officials. Certain studies reportedly took body samples of children with NS, including blood, skin snips, urine samples, and brains for autopsies. Some children were also reported to have been taken to the U.S.A. for further research. For community members, all this research, perceived as invasive, has not produced concrete results on the aetiology of the disease, which leads to feelings of anxiety, research fatigue and suspicions around the motives of the foreign researchers. The reported lack of dissemination of results lead to bits and pieces of information being shared in the form of gossip and rumours. People further perceived the lack of dissemination as purposefully withheld information and reinforced the belief that foreign researchers were simply using their children’s body parts for profits. Complaints were present across all study villages without exception i.e. researchers came, took information and never returned with results. This has led to research fatigue, distrust and desperation about finding a cure or answer. “*I wonder if researchers are just making money with our children’s samples*”. (IDI, mother of NS child)

As a consequence of the lack of dissemination in addition to the absence of concrete results, people stated their intention to refuse co-operation in further research. Suspicions about the government’s supposed ill-intentions towards the Acholi were increasing, limited dissemination further reinforced their suspicions of researchers and the government purposefully withholding information to cover up their involvement. Box 3 illustrates how research results were being disseminated as gossip, which created an opportunity for rumours to be spread and alternative interpretations to be mooted. Similarly, inadequately planned dissemination as depicted in Box 4, proved futile and created even more confusion and distrust in the community.

**Box 3. *Ambiguity and rumours regarding research results lead to suspicions towards foreigners***

At the funeral of one of the girls who had suffered from NS, a local political official who was seemingly very passionate about safeguarding her community, addressed the congregation saying “*Many Americans have conducted research on this disease and around August or September this year (2015) some Americans who did tests and check-ups on these children came back and said that they found some kind of blisters in the children’s brains*. *So*, *we think that this is some kind of planned sickness that whites make to get profit*.” She also added that the district officials did not like her and the village health worker because they spoke the truth about this disease.

When we looked more into these rumours regarding ‘blisters’ in the brain, a person from the ministry of health explained that it was true that some crystal-like formations had been seen in the brain autopsies of NS children, however there was an ongoing debate amongst the scientific community about whether these crystals were simply an artefact of long and poor storage of samples and questioned whether they were linked to the cause or aetiology of the disease as there had been reported delays in the samples being shipped to the Unites States. Due to the uncertainty about cause of these crystal-like formations, nothing could be reasonably disseminated to the people in the affected communities. The challenge here was that partial knowledge was being disseminated by unqualified members of the community thereby adding to the suspicions and distrust.

**Box 4. Poor preparedness before dissemination**

The lack of community engagement, but also the pre-mature dissemination of research findings among community members can generate rumours, confusion, frustration and research fatigue, as well as widening the knowledge gap. This was seen at the 2^nd^ international conference on NS in Gulu, held in July 2015. Many clinicians, neurologists and researchers had heard complaints about the lack of dissemination, and so during one field visit to Tumangu village during the conference, where several parents of affected children were gathered, scientists spontaneously decided to address the community and share results. Dissemination in this way was unplanned, and epidemiological and medical vocabulary that would not make sense to a lay person was used, for example, “a case-control study showed an association to onchocerciasis”. A local health professional spontaneously attempted to translate the scientists’ message into Acholi. This ‘dissemination’ was followed by a slew of questions from parents and carers of those affected who were seemingly confused whether it was the blackfly causing the illness or not and what they should do to cure their children.

In 2017, the community distrust was also expressed when we asked about a planned dissemination by the MoH, which took place in December 2016. An aggregate of all research findings was reportedly compiled and village leaders were invited to attend. One village leader who attended the dissemination stated, “*Yes*, *last year (2016) a dissemination meeting was held in Pader (district) by people from the ministry for both Lango and Acholi sub region*. *They invited us and I went but we don’t believe those findings*. *I wasn’t very interested because I think that meeting was meant for making money for Christmas since Christmas was just around the corner*. *They told us this (NS) was happening because of the blackfly but we don’t believe them because blackflies have always been here; so why didn’t this condition come up then*? *I think NS is happening because of the war because the places heavily affected are places where most killings happened*. *And why is it only in Northern Uganda and not in Western (where the current leader comes from)*?”

## Discussion

Nodding Syndrome prevention and treatment in Northern Uganda have become central to the country’s political agenda [46]. While disease control, as such, always has a political dimension, the multiple levels of ‘ambiguity’ of Nodding Syndrome has direct implications for further research and the control of this disease. Disease conceptions for NS, similar to epilepsy, are not fixed but constantly created and recreated from particular perspectives [40]. The unknown aetiology of NS creates an opportunity for different avenues for reasoning and rationalising for community members, health workers and researchers alike.

Among the Acholi in Northern Uganda this has contributed to distrust in government interventions and research projects alike, giving rise to rumours of secrecy and hidden agendas of the government against the Acholi people. This contributes to a challenging situation: if the government does not intervene, it is seen as neglect; and when it does, people are suspicious of the aid and the motives behind it. The war and perceptions of neglect and powerlessness have led to annihilation anxieties among the Acholi, which is a common phenomenon in authoritarian socio-political contexts [42, 45]. As with NS, other epidemics have reflected these regional and ethnic tensions. During the Ebola Virus Disease (EVD) epidemic in 2000, also occurring during the LRA war, the Acholi attributed the first case to the government intentionally planting the disease in order to harm them. This was reportedly done by the government when an infected corpse of a deceased Acholi soldier was sent to his family in the north, despite the government’s awareness about the Acholi tradition of washing and preparing the corpse for burial [32]. The Acholi believed that the government knew that touching the corpse would spread the disease but they sent the corpse to them anyway. These suspicions and fears were confirmed when the president did not express remorse for the Acholi lives lost from EVD [32]. Similarly, the HIV epidemic amongst the Acholi was also explained as an intentional effort by the government to spread the disease. Both men and women were reportedly raped by infected government soldiers from other regions in order to spread the disease [43]. The NS epidemic, similarly, coincided with the LRA war against the ruling government. Government response to the outbreak was slow and when it finally occurred it was driven, and continues to be driven, by politicians and made part of the political agenda. The Acholi’s suspicions of the government and its prevention, treatment and research interventions are further fed by their sense of desperation and poverty and limited economic progress. Enduring decades of war, Northern Ugandans have shown resilience but the trauma from it has been embodied as distrust towards the national leadership and so a defensive stance towards initiatives made by the government is taken. The consequences of structural violence, political economy and poverty as embodied in ill-health and trauma are described in several texts [32, 43, 44, 47, 48]. The added fear of being victimised is overshadowed with hopelessness [49].

Another contribution to this distrust is the perceived lack of proper dissemination of research results. The ambiguity around aetiology, delayed dissemination, inconclusive results and lack of a cure for the disease, despite years of research and invasive collection of body samples, contributes to make dissemination a real challenge. The other challenge regarding dissemination is that research findings take time to analyse and may be published years after the data was collected. Research findings are generally published a few years after the data collection was conducted and by the time results are published most project funds have been spent and the research projects have ended. Such delays due to the structure of research projects and their funding are extremely problematic. It is crucial for funds to be kept for the dissemination of the research among locally affected populations at the end of the study—ethical committees and the Ministry of Health should make this an obligation.

The general distrust of government motives can also lead people to dismiss all health interventions, including mass drug administration of IVM. This has not posed as a problem for onchocerciasis control yet, as Uganda has had a very strong vector control program since 2012 where breeding sites of blackflies in rivers have regularly been treated with larvicides [25], thus reducing blackfly populations that transmit the OV parasite. Adam Hendy, an entomologist who worked in the area from 2014 to 2016 also confirmed that blackfly populations were very low. However, if for some reason, blackfly populations increase and onchocerciasis control is dependent solely on IVM distribution, this can become a public health problem in Northern Uganda.

Feelings of distrust were not limited to the government, but also directed towards foreigners, researchers, and Euro-Americans. The notion that foreigners came to Africa to harvest body parts for profit has been another long-standing belief, similar to rumours of blood stealing in Sub Saharan Africa [45, 50]. One example of such beliefs was documented during the EVD epidemic when individuals were taken to isolation units for treatment. The treatment units were isolated with tarp fences, preventing relatives from visiting or seeing patients inside the unit. If the patients died, medical safety protocol required that the bodies were instantly placed in a corpse bag and shifted to a burial ground near the airport. The protocol for such emergencies did not foresee for the family to be notified, nor were they allowed to see the body, which fed rumours [32]. Inadequate community engagement in the protocol was experienced as secrecy from the perspective of the locals, amplifying feelings of suspicion towards international response teams [32].

While NS control in Northern Uganda is complex and multi-layered, a consistent, continuous and clear response by the government and researchers would contribute to limiting the ambiguity surrounding NS and its interpretations. The government’s response has been inconsistent and driven by media attention and political propaganda to maintain political control and economic power. Top-down approaches to research with minimal community engagement and dissemination gives rise to more rumours and misinformation [42, 51], which has exacerbated the mistrust and set up frameworks for the failure of future interventions in Northern Uganda.

Government support needs to be comprehensive and consistent for trust to be built up again. This data has shown how crucial it is for the government and for international agencies to take into consideration the cultural context of locals and perceptions regarding the causes of NS. Lack of continuous information and engagement will fuel distrust. To intervene effectively, at least some of the ambiguity and uncertainty must be overcome. The first step would be to engage the community actively throughout the different stages of research.

## Strengths and limitations of the study

Conducting an ethnographic study over a three-year period and use of multiple qualitative methods of data collection facilitated an in-depth exploration of NS, context, interventions and gaps in relation to community engagement in research and programming including how these fit within the politically sensitive Northern Uganda. Our findings should be interpreted in view of the following limitations:

The qualitative nature of the study does not facilitate quantification of community perceptions and practices. However, use of multiple methods of data collection helped to triangulate our findings. The study covered only two districts that were affected by war, thus applicability of our findings to other areas especially those not affected by war may be limited. Additionally, it is possible that people from Kitgum district have politicized Nodding Syndrome more than other districts as the parliamentarian who first brought the attention in the media in 2012 about Nodding Syndrome came from and represented Kitgum district. Additionally, to strengthen the understanding of how politicization affects health interventions in particular communities, it would have been valuable to conduct fieldwork in another onchocerciasis-endemic area of Uganda where a different ethnic group was in majority and where they were not subjected to war and civil conflict, e.g. Masindi district in Western Uganda as comparison. However, inclusion of highly affected and less affected areas in this study increases possibilities for generalizability of our findings.

### Conclusion and recommendations

Given the ambiguity around NS, and the political context in Northern Uganda, moving forward NS requires the building of trust, the de-politicization of response, and more clarity and transparency. Trust can be built by engaging the community and making them active participants in every stage of the intervention, be it research, service delivery for treatment and prevention, and sensitisation. In addition, community participatory implementation and research methodologies should be used that include continuous community involvement and feedback loops to address rumours and feelings of resentment. This is best achieved through the extended involvement of the community including day-to-day interactions with implementation staff. By creating continuous feedback loops, the sharing of information and engaging in active dialogue, ambiguities around treatment, prevention and the intention of the research will be reduced. Dissemination must be planned. Well-prepared sessions on interim research findings must be conducted at regular intervals despite inconclusive results. This could be a requirement from the Ugandan Ethics Committees and the national Council for Science and Technology for every research, particularly on NS.
